# Datasets for the reporting of primary tumour in bone: recommendations from the International Collaboration on Cancer Reporting (ICCR)

**DOI:** 10.1111/his.14849

**Published:** 2022-12-20

**Authors:** Judith V M G Bovée, Fleur Webster, Fernanda Amary, Daniel Baumhoer, J L (Hans) Bloem, Julia A Bridge, Justin M M Cates, Enrique de Alava, Angelo Paolo Dei Tos, Kevin B Jones, Annabelle Mahar, G Petur Nielsen, Alberto Righi, Andrew J Wagner, Akihiko Yoshida, Christopher D M Fletcher

**Affiliations:** ^1^ Department of Pathology Leiden University Medical Center Leiden The Netherlands; ^2^ Leiden Center for Computational Oncology, LUMC Leiden The Netherlands; ^3^ International Collaboration on Cancer Reporting Sydney NSW Australia; ^4^ Department of Histopathology Royal National Orthopaedic Hospital Stanmore Greater London UK; ^5^ Cancer Institute, University College London London UK; ^6^ Bone Tumour Reference Centre Institute of Pathology, University Hospital Basel Basel Switzerland; ^7^ Department of Radiology Leiden University Medical Center Leiden The Netherlands; ^8^ Division of Molecular Pathology ProPath Dallas TX USA; ^9^ Department of Pathology and Microbiology University of Nebraska Medical Center Omaha NE USA; ^10^ Department of Pathology Vanderbilt University Medical Center Nashville TN USA; ^11^ Institute of Biomedicine of Sevilla (IBiS), Virgen del Rocio University Hospital, CSIC, University of Seville Seville Spain; ^12^ Department of Normal and Pathological Cytology and Histology School of Medicine, University of Seville Seville Spain; ^13^ Department of Pathology Azienda Ospedaliera Universitaria di Padova Padova Italy; ^14^ Department of Medicine University of Padua, School of Medicine Padua Italy; ^15^ Department of Orthopaedics Huntsman Cancer Institute, University of Utah School of Medicine Salt Lake City UT USA; ^16^ Department of Oncological Sciences Huntsman Cancer Institute, University of Utah School of Medicine Salt Lake City UT USA; ^17^ Department of Tissue Pathology and Diagnostic Oncology Royal Prince Alfred Hospital Camperdown NSW Australia; ^18^ Department of Pathology Massachusetts General Hospital Boston MA USA; ^19^ Harvard Medical School Boston MA USA; ^20^ Department of Pathology IRCCS Istituto Ortopedico Rizzoli Bologna Italy; ^21^ Center for Sarcoma and Bone Oncology Dana‐Farber Cancer Institute Boston MA USA; ^22^ Department of Diagnostic Pathology National Cancer Center Hospital Tokyo Japan; ^23^ Rare Cancer Center National Cancer Center Hospital Tokyo Japan; ^24^ Department of Pathology Brigham & Women's Hospital, Harvard Medical School Boston MA USA

**Keywords:** bone sarcoma, data management, data registration, dataset, guidelines, ICCR, structured report, synoptic report

## Abstract

**Background and objectives:**

Bone tumours are relatively rare and, as a consequence, treatment in a centre with expertise is required. Current treatment guidelines also recommend review by a specialised pathologist. Here we report on international consensus‐based datasets for the pathology reporting of biopsy and resection specimens of bone sarcomas. The datasets were produced under the auspices of the International Collaboration on Cancer Reporting (ICCR), a global alliance of major (inter‐)national pathology and cancer organisations.

**Methods and results:**

According to the ICCR's process for dataset development, an international expert panel consisting of pathologists, an oncologic orthopaedic surgeon, a medical oncologist, and a radiologist produced a set of core and noncore data items for biopsy and resection specimens based on a critical review and discussion of current evidence. All professionals involved were bone tumour experts affiliated with tertiary referral centres. Commentary was provided for each data item to explain the rationale for selecting it as a core or noncore element, its clinical relevance, and to highlight potential areas of disagreement or lack of evidence, in which case a consensus position was formulated. Following international public consultation, the documents were finalised and ratified, and the datasets, including a synoptic reporting guide, were published on the ICCR website.

**Conclusion:**

These first international datasets for bone sarcomas are intended to promote high‐quality, standardised pathology reporting. Their widespread adoption will improve the consistency of reporting, facilitate multidisciplinary communication, and enhance comparability of data, all of which will help to improve management of bone sarcoma patients.

## Graphical abstract

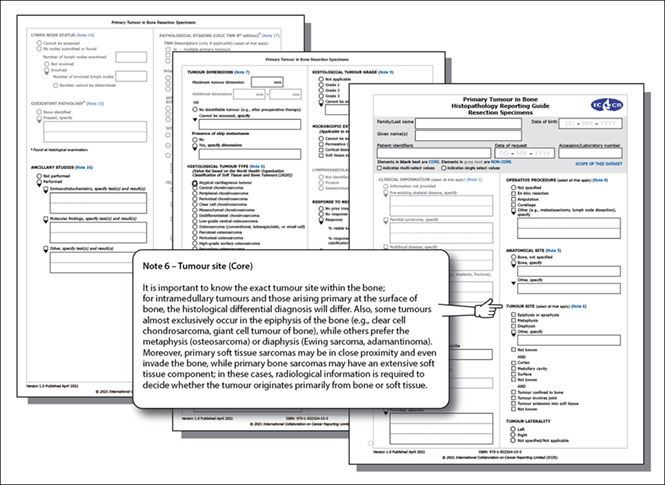

## Introduction

Pathology reporting on cancer resection specimens provides information that is essential for individual patient management, used for clinical trials and tissue‐based research, and recorded in cancer registries. Given this central role of pathology data in cancer care and research at both the individual and population levels, standardised and structured pathology reporting is essential to ensure that the relevant information is complete, unambiguous, and delivered in a user‐friendly format.

Evaluation of bone tumour biopsies is often perceived as highly challenging by pathologists because of their rarity, the relatively high number of distinct tumour subtypes (which often show overlapping histomorphology), and the requirement for clinical‐radiological correlation to come to an accurate diagnosis. Moreover, surgical resection specimens can be complex to evaluate/process due to the various anatomic locations that may be involved and the necessity for extensive macroscopic evaluation, documentation, and correlation with imaging findings. Thus, for accurate diagnosis of bone tumours a multidisciplinary approach is imperative. It is the responsibility of the clinician or radiologist requesting the pathological examination of a specimen to provide information to the pathologist that will assist subsequent tissue processing, diagnostic evaluation, and final interpretation. The use of a standardised pathology requisition/request forms including a checklist of important clinical information is strongly encouraged to help ensure that these data are provided by submitting clinicians. It is the responsibility of the pathologist to verify that all radiological and clinical information essential to make a diagnosis is available to guarantee that the final diagnosis is made within the appropriate clinical/imaging context. This is often achieved through discussion at a multidisciplinary tumour board meeting.

Several worldwide organisations such as the College of American Pathologists (CAP) and the Royal College of Pathologists (RCPath) have independently developed datasets for pathology reporting on bone sarcoma.^1^, ^2^, ^3^ The International Collaboration on Cancer Reporting (ICCR) coordinates the production of evidence‐based international pathology reporting datasets that have a consistent style and contain all the parameters needed to guide patient management. The ICCR is a collaboration of multiple pathology organisations and has alliances with international cancer organisations, including the International Agency for Research on Cancer (IARC), Union for International Cancer Control (UICC), and American Joint Committee on Cancer (AJCC). The ICCR datasets are freely available from the ICCR website (http://www.iccr‐cancer.org).

Here we report on the development of datasets for the pathology reporting of primary bone sarcomas (both biopsy and resection specimens), discuss the rationale for the inclusion of data items, and propose a consensus position in areas of controversy and where there is limited evidence to assist pathologists in their diagnostic practice.

## Materials and Methods

The ICCR has developed a set of standardised operating procedures for the process of dataset development and has also defined the selection process, roles, and responsibilities of the chair, expert panel members, the ICCR Dataset Steering Committee representative(s) on the panel, ICCR Series Champion, and the project manager (http://www.iccr‐cancer.org/datasets/dataset‐development). The ICCR Series Champion provided guidance and support to the Chair of the Dataset Authoring Committee (DAC) regarding ICCR standards and ensured harmonisation across the bone and soft‐tissue suite of datasets. An international expert panel consisting of pathologists, an oncologic orthopaedic surgeon, a medical oncologist, and a radiologist was established. Initial draft documents were produced by the Project Manager and chair after assessment of core and noncore data items within existing international datasets for bone sarcomas. These drafts were circulated to the Dataset Authoring Committee (DAC) and individual dataset items were discussed at a coordinated series of teleconferences. Subsequently, an agreed version of the revised datasets was posted for open international consultation on the ICCR website for a period of 2 months. All comments received were subsequently discussed by the DAC and, where there was universal agreement from DAC members, resultant changes were incorporated into the datasets. Final versions were ratified by the ICCR Dataset Steering Committee prior to publication. All ICCR datasets, including these on bone sarcomas, are freely available worldwide at the ICCR website at www.iccr‐cancer.org/datasets.

## Results

### Scope

The ICCR has developed two separate datasets for the pathology reporting of biopsy and resection specimens of primary bone tumours. Ewing sarcoma and related round‐cell sarcomas arising in bone are also covered in these datasets. Some types of soft‐tissue sarcoma may on rare occasion arise primarily in bone and should be reported using the primary tumour in bone datasets, rather than the soft‐tissue sarcoma datasets. If biopsies are taken from multiple tumour nodules at different sites, these should be documented separately. Haematologic malignancies and metastatic specimens were excluded from these datasets.

### Core elements

Core elements are those that are essential for the clinical management, staging, or prognosis of the cancer. These elements will either have evidentiary support at Level III‐2 or above (based on prognostic factors in the National Health and Medical Research Council (NHMRC) levels of evidence^4^). The summation of all core elements is considered the minimum reporting standard.

A summary of the core elements for the biopsy and resection datasets is outlined in Tables 1 and 2, respectively, and each is described in further detail below.

**Table 1:** Core and noncore elements for the pathology reporting of primary tumour in bone biopsy specimens

Core	Noncore
Imaging findings	Clinical information
Operative procedure Type	Operative procedure Number of cores
Histological tumour type	Biopsy handling
Histological tumour grade	Necrosis
Ancillary studies	Lymphovascular invasion
	Coexistent pathology

**Table 2:** Core and noncore elements for the pathology reporting of primary tumour in bone resection specimens

Core	Noncore
Neoadjuvant therapy Type	Clinical information
Imaging findings	Neoadjuvant therapy Included in clinical trial
Operative procedure	Tumour dimensions Additional dimensions
Anatomical site	Lymphovascular invasion
Tumour site	Margin status Type of tissue of closest margin Distance of tumour to osteotomy (if not the closest margin)
Tumour laterality	Lymph node status
Tumour dimensions Largest diameter Presence of skip metastases	Coexistent pathology
Histological tumour type	Pathological staging
Histological tumour grade	
Microscopic extent of invasion	
Margin status R0 R1 R2 Distance to and localisation of closest margin	
Ancillary studies	

### Neoadjuvant therapy

For resection specimens, information about neoadjuvant treatment is essential for proper interpretation of the microscopic findings and accurate pathological diagnosis. Preoperative radiation and/or other therapy may have a profound effect on the morphology of both the cancer and benign tissue. Knowledge of such prior therapy may help to interpret changes such as necrosis, cellular atypia, and inflammatory infiltrates. For this reason, information regarding any prior therapy is important for the accurate assessment of bone specimens. Different scoring systems are being used and are discussed under ‘Response to neoadjuvant therapy’. For example, the use of denosumab in giant‐cell tumour of bone induces bone formation and reduces the number of multinucleated osteoclast‐like giant cells within the lesion; therefore, this information is crucial for diagnostic interpretation. Also, previous embolisation may cause areas of necrosis. Moreover, neoadjuvant use of many novel therapies (such as tyrosine kinase inhibitors or immunotherapy) may result in histological effects and need to be fully disclosed.

### Imaging findings

Correlation between histologic and radiologic findings is critical in the diagnosis of bone tumours. Ideally, every case should be discussed in a multidisciplinary conference or the pathologist should have at least access to the imaging findings when evaluating a biopsy. This is the main reason imaging findings are considered a core element in bone tumour evaluation. For instance, aggressive features identified radiographically (permeative/moth‐eaten growth, cortical destruction, soft tissue extension, type of periosteal reaction) should be mentioned here, as well as multifocality, evidence of matrix deposition, presence of fluid–fluid levels, etc. For instance, in cartilaginous tumours in the phalanx or in Ollier disease, the distinction between benign and malignant may depend solely on whether there is cortical destruction, which may be impossible to evaluate on biopsy or fragmented curettage specimens alone. Therefore, these diagnoses cannot be made without radiological correlation. The presence of fracture should always be documented, as it may alter the morphological features and, in some instances, simulate aggressive features, such as host bone entrapment. As the histological alterations caused by the fracture change over time, it is important to know the time frame between fracture and biopsy. Finally, certain bone tumours (cartilaginous tumours, vascular tumours) tend to occur multifocally, and this information is also helpful for the pathologist. The histological diagnosis should always be correlated with the radiological diagnosis and one should always be cautious when there is a discrepancy between radiological and histological findings. Multidisciplinary discussion is essential, and repeat biopsy should be considered if differences of opinion are not resolved.

It is important to know the exact tumour site within the bone, since the histological differential diagnosis will differ between intramedullary tumours and those arising primarily at the bone surface. Also, some tumours are almost exclusively found in the epiphyseal region (e.g. clear‐cell chondrosarcoma, giant‐cell tumour of bone, chondroblastoma), while others preferentially affect the metaphysis (osteosarcoma) or involve also the diaphysis (Ewing sarcoma, adamantinoma). Moreover, primary soft‐tissue sarcomas may arise adjacent to and even invade bone, while primary bone sarcomas may have an extensive soft‐tissue component. In these cases, radiological information is required to decide whether the tumour originates primarily from bone or soft tissue.

It is also important for the pathologist to be aware of the radiological differential diagnosis when evaluating bone resection specimens. The presence of a pathologic fracture may influence histological evaluation and should be documented. Certain bone tumours (cartilaginous tumours, vascular tumours) tend to occur multifocally, and skip metastases can be present. This is important information for the pathologist when working up the resection specimen. Finally, the radiological response evaluation should be recorded after neoadjuvant therapy.

### Anatomical site

For biopsy specimens, the anatomical site should be documented by the radiologist and reported under ‘Imaging findings’. Recording the anatomical site of the tumour is important, since certain bone tumours have predilections to arise in specific bones and not others, and/or there is a strong association between anatomic site and patient outcome. The latter is especially true for cartilaginous tumours; as a consequence, the World Health Organization (WHO) Classification of Tumours, Soft Tissue and Bone Tumours (5th edition, 2020)^5^ distinguishes between atypical cartilaginous tumour and chondrosarcoma grade 1, depending on whether the tumour is located in the appendicular or axial skeleton, respectively. When arising at appendicular sites (the long and short tubular bones), these tumours behave in a locally aggressive manner and do not metastasize. Therefore, they can be treated locally and should not be classified as having full malignant potential. The term ‘atypical cartilaginous tumour’ is thus preferred for cartilaginous tumours involving the long and short tubular bones. In contrast, the term ‘chondrosarcoma, grade 1’ is used for histologically similar tumours of the axial skeleton, including the pelvis, scapula, and skull base (flat bones)―reflecting the poorer clinical outcome and the necessity for more aggressive treatment of these tumours at these sites.

It should be noted that the definition of axial versus appendicular is not universally accepted; while the 2020 WHO Classification^5^ categorizes the scapula and skull base as part of the axial skeleton, the UICC^6^/AJCC^7^ TNM 8th editions include these sites with the appendicular skeleton. Here we consider the scapula and skull base to be part of the axial skeleton.

### Tumour site

For biopsy specimens, the exact tumour site within the bone should be documented by the radiologist and reported under ‘Imaging findings’.

### Tumour laterality

For biopsy specimens, laterality should be documented by the radiologist or clinician and is reported under ‘imaging findings’. Tumour laterality is a core element.

### Tumour dimensions

For biopsies, the size of the largest tumour nodule should be documented by the radiologist based on imaging, preferably in three dimensions, as this is important to evaluate the tumour volume; in the dataset this will be reported under ‘imaging findings’. In cases where the radiological tumour dimensions cannot be assessed, such as for discontinuous tumour, it is important to note this and record the volume of tumour if possible. If biopsies are taken from multiple tumour nodules at different sites, these should be documented separately.

When reporting the gross evaluation of a bone resection specimen, the pathologist should measure the size of the tumour on the resection specimen in at least its largest linear dimension (core element), but preferably in three dimensions (noncore element), as this is important in estimating tumour volume.

### Operative procedure

This element includes the type and intent of the operative procedure, independent of the final margin assessment by the pathologist. On the rare occasion that lymph nodes are included with the specimen, these should be listed under ‘other’. Metastasectomy specimens can also be listed under ‘other’.

### Histological tumour type

Histologic diagnosis is based on the WHO classification of soft tissue and bone tumours, 5th edition, 2020 (Table 3). The diagnosis is usually made on biopsy before resection. In some cases, the biopsy is suboptimally targeted on the area(s) of interest or affected by the surgical process, leaving the pathologist with tissue that can be underrepresentative or misrepresentative of the lesion based on the imaging studies. For some entities, more sophisticated testing (e.g. molecular analysis) may be required to achieve an accurate diagnosis, but the small tissue size, tissue processing issues, or suboptimal targeting of biopsy materials may preclude ancillary diagnostic testing. The pathologist should specify any and all limitations of the tissue sample that prevent achieving an optimal pathologic diagnosis. In addition, comments can be made in case the diagnosis on biopsy is uncertain for reasons other than limitations of the material or when there remains a differential diagnosis. When reporting resection specimens, a comment should be included if the final diagnosis based on the resection specimen is discordant with the previous diagnosis on the biopsy.

**Table 3:** World Health Organization classification of intermediate and malignant bone tumours and undifferentiated small round‐cell sarcomas^5^

Descriptor	ICD‐O codes^a^
Chondrogenic tumours	
Intermediate (locally aggressive)	
Atypical cartilaginous tumour	9222/1
Malignant	
Chondrosarcoma, grade 1	9222/3^b^
Chondrosarcoma, grade 2	9220/3
Chondrosarcoma, grade 3	9220/3
Periosteal chondrosarcoma	9221/3
Clear cell chondrosarcoma	9242/3
Mesenchymal chondrosarcoma	9240/3
Dedifferentiated chondrosarcoma	9243/3
Osteogenic tumours	
Malignant	
Low‐grade central osteosarcoma	9187/3
Osteosarcoma	9180/3
Conventional osteosarcoma	
Telangiectatic osteosarcoma	
Small cell osteosarcoma	
Parosteal osteosarcoma	9192/3
Periosteal osteosarcoma	9193/3
High‐grade surface osteosarcoma	9194/3
Secondary osteosarcoma	9184/3
Fibrogenic tumours	
Malignant	
Fibrosarcoma NOS	8810/3
Vascular tumours of bone	
Malignant	
Epithelioid haemangioendothelioma NOS	9133/3
Angiosarcoma	9120/3
Osteoclastic giant cell–rich tumours	
Intermediate (locally aggressive, rarely metastasizing)	
Giant cell tumour of bone	9250/1
Malignant	
Giant‐cell tumour of bone, malignant	9250/3
Notochordal tumours	
Malignant	
Conventional chordoma	9370/3
Chondroid chordoma	
Poorly differentiated chordoma	9370/3
Dedifferentiated chordoma	9372/3
Descriptor	ICD‐O codes^a^
Other mesenchymal tumours of bone	
Malignant	
Adamantinoma of long bones	9261/3
Dedifferentiated adamantinoma	
Leiomyosarcoma NOS	8890/3
Pleomorphic sarcoma, undifferentiated	8802/3
Haematopoietic neoplasms of bone	
Plasmacytoma of bone	9731/3
Malignant lymphoma, non‐Hodgkin, NOS	9591/3
Hodgkin disease NOS	9650/3
Diffuse large B‐cell lymphoma NOS	9680/3
Follicular lymphoma NOS	9690/3
Marginal zone B‐cell lymphoma NOS	9699/3
T‐cell lymphoma NOS	9702/3
Anaplastic large cell lymphoma NOS	9714/3
Malignant lymphoma, lymphoblastic, NOS	9727/3
Burkitt lymphoma NOS	9687/3
Langerhans cell histiocytosis NOS	9751/1
Langerhans cell histiocytosis, disseminated	9751/3
Erdheim–Chester disease	9749/3
Rosai–Dorfman disease	
Undifferentiated small round cell sarcomas	
Ewing sarcoma	9364/3
Round cell sarcoma with *EWSR1*–non‐ETS fusions	9366/3^b^
*CIC*‐rearranged sarcoma	9367/3^b^
Sarcoma with *BCOR* genetic alterations	9368/3^b^

© World Health Organization/International Agency for Research on Cancer. Reproduced with permission.

^a^ These morphology codes are from the International Classification of Diseases for Oncology, third edition, second revision (ICD‐O‐3.2). Behaviour is coded /0 for benign tumours; /1 for unspecified, borderline, or uncertain behaviour; /2 for carcinoma in situ and grade III intraepithelial neoplasia; /3 for malignant tumours, primary site; and /6 for malignant tumours, metastatic site.

^b^ Codes marked with an asterisk were approved by the International Agency for Research on Cancer/World Health Organization Committee for ICD‐O at its meeting in January 2020. Incorporates all relevant changes from the 5^th^ Edition Corrigenda October 2020.

### Histological tumour grade

In bone sarcomas, the histotype primarily determines histologic grade (based on the 2020 WHO Classification^5^), with only very few exceptions. Bone sarcomas in which the grade is determined by histotype are outlined in Table 4.

**Table 4:** Bone sarcomas in which grade is determined by histotype

Grade 1(low‐grade)	Low‐grade central osteosarcoma Parosteal osteosarcoma Clear cell chondrosarcoma
Grade 2 (intermediate‐grade)	Periosteal osteosarcoma
Grade 3 (high‐grade)	Osteosarcoma (conventional, telangiectatic, small cell, secondary, high‐grade surface) Undifferentiated high‐grade pleomorphic sarcoma Ewing sarcoma and BCOR‐rearranged sarcoma Dedifferentiated chondrosarcoma Mesenchymal chondrosarcoma Dedifferentiated chordoma Poorly differentiated chordoma Angiosarcoma
Variable	Conventional chondrosarcoma (grade 1–3 according to Evans)^5^, ^8^ Leiomyosarcoma of bone (Grade 1–3 no established grading system) Low‐ and high‐grade malignancy may occur in giant cell tumour of bone
Not applicable	Adamantinoma and conventional chordoma

### Microscopic extent of invasion

For correlation with imaging findings, histological evidence of permeative growth, cortical invasion, and destruction or soft tissue extension should be recorded when reporting resection specimens. This is facilitated when gross examination is aligned with the radiological imaging. Thus, preferably radiologic images should be available when processing specimens.

### Response to neoadjuvant therapy

The response to preoperative chemotherapy is of prognostic value, especially in Ewing sarcoma and osteosarcoma, and needs to be evaluated in a standardised way when reporting resection specimens. At least one complete central slab of tumour through its largest dimension should be submitted for histological evaluation. Additional sections can be taken from the remaining two hemispheres of the specimen, especially near the periosteum and/or areas of soft tissue extension. The amount of remaining viable tumour cells should be estimated on each histological slide to obtain an average score reflecting the overall percentage of response. Response does not always consist of necrosis; very often, extensive fibrosis and calcification can be seen, which is also considered response. In osteosarcoma, a cutoff of 10% viable tumour cells (or 90% or more response consisting of tumour necrosis, fibrosis, and calcification) is used to indicate a good response.^9^ For Ewing sarcoma, the cutoff is less well defined. Albergo *et al*. (2016) recently showed that a 100% response was optimal to define a good tumour response in Ewing sarcoma.^10^ In earlier reports (the Bologna system^11^ as well as the van der Woude scoring system^12^), a good response was defined as the percentage of necrosis of the microscopic tumour mass between 90% and 100%. In the literature, different cutoffs are used to evaluate chemotherapy‐induced necrosis.^13^, ^14^, ^15^, ^16^

### Margin status

Most features relating to the margin status of resection specimens are core (Table 2). There is no generally accepted approach for reporting bone tumour margins. If margins are involved, a distinction is often made between microscopic involvement (R1) and resections in which it is evident macroscopically that the tumour has been incompletely resected (R2). In case of negative margins (R0), the minimum that should be documented is the distance of the tumour to the closest margin. Some guidelines recommend that all margins <20 mm should be documented in terms of depth and the tissue comprising each that is <20 mm (e.g. fascia, periosteum, epineurium, vascular sheath).

### Ancillary studies

All immunohistochemical stainings and molecular tests that contributed to the diagnosis should be documented. For instance, for Ewing sarcoma and other round‐cell sarcomas, lymphoma, adamantinoma, and chordoma, these ancillary studies (immunohistochemical and/or molecular) are critical.

### Noncore elements

Noncore elements are those which were unanimously agreed by the committee to be included in the dataset but are not supported by NHMRC level III‐2 evidence.^4^ These elements may be clinically important and recommended as good practice but are not yet validated or regularly used in patient management. A summary of the noncore elements for each of the datasets is outlined in Tables 1 and 2 and each is described below.

### Clinical information

For accurate diagnosis of bone tumours, a multidisciplinary approach is imperative. It is the responsibility of the clinician or radiologist requesting the pathological examination of a specimen to provide information to the pathologist that will have an impact on the diagnostic process or affect its interpretation. The use of a standard pathology requisition/request form including a checklist of important clinical information is strongly encouraged to help ensure that this information is provided by the clinicians with the specimen.

It is also the responsibility of the pathologist to verify that all radiological and clinical information essential to make a diagnosis is available to guarantee that the final diagnosis is made within the appropriate clinical/imaging context. This is often achieved through discussion at a multidisciplinary tumour board meeting.

### Biopsy handling

Core needle biopsy is often performed under computed tomography (CT) or ultrasound guidance with all imaging studies available for review during the planning and execution of the procedure. Preferably a minimum of three cores are submitted for diagnosis. A frozen section can be performed on a representative selection of cores or the tissue obtained at open biopsy, to evaluate whether the biopsy has yielded adequate tissue for diagnosis. Adequacy may also be determined by cytological rapid on‐site evaluation (ROSE); the advantage of ROSE is that the biopsy core evaluated remains almost entirely intact, preserving tissue for other ancillary testing. Moreover, a provisional diagnosis can sometimes be given, and based on the results the remaining tissue can be triaged for further work‐up. Bone tumours need decalcification when formalin‐fixed and paraffin‐embedded (FFPE), which, depending on the type of decalcification used, may severely hamper the use of ancillary techniques. Decalcification should optimally be performed with solutions that preserve RNA and DNA, or a representative core should be kept frozen or embedded in paraffin without prior decalcification, to allow for molecular testing. Acid‐based decalcification (other than EDTA) should therefore be avoided if frozen tissue is unavailable.

### Necrosis

Necrosis in biopsy specimens where the patient has not received neoadjuvant treatment should be documented, especially if necrosis is abundant, hampering microscopic evaluation of the tumour.

### Lymphovascular invasion

Lymphovascular invasion (LVI) is extremely rare in bone tumours. However, it is important to report if identified in the specimen.

### Margin status

In addition to documentation of involvement of margins (R0, R1, R2, distance of tumour from closest margin and localisation of the closest margin) which are considered core, some additional features of margin status are noncore (Table 2). The type of tissue comprising the resection margin could also be recorded (e.g. pseudocapsule, loose fibrous/fibroadipose tissue, bone, skeletal muscle, dense regular connective tissue fascia/aponeurosis/periosteum/vascular sheath/perineurium) since bone and fascia may be more robust marginal tissues than other tissue types. In addition, the distance to the closest osteotomy margin could also be recorded even if it is not the closest margin.

### Lymph node status

Lymph nodes are very rarely submitted or found with bone specimens and it is not necessary to undertake an exhaustive search for nodes in the specimen. Although regional lymph node metastasis is very rare in adult bone sarcomas, its presence has prognostic importance and it is important to report.

### Coexistent pathology

If present, the pathologist should report other abnormalities that are relevant for the diagnosis and any other significant pathologic finding, even if unrelated or not directly relevant. For instance, the presence of precursor lesions for chondrosarcoma, such as multiple enchondromas, osteochondromas, or the presence of synovial chondromatosis, should be documented. Paget disease, osteonecrosis, or bone infarction may be seen in association with a secondary sarcoma. The presence of a pathologic fracture may influence the histological evaluation and should be documented. Other unrelated findings may include vasculitis, infection, coexistent chronic lymphocytic leukaemia (CLL), or incidental/unexpected metastatic carcinoma in the same specimen.

### Pathological staging

It is important that pathologists document the required parameters for tumour staging (according to UICC^6^ or AJCC^7^ 8th edition Staging Systems) in their reports. Ultimately, the final stage will be determined by the treating physician or by the multidisciplinary team, which will take both the pathological and imaging findings into account.

## Discussion

Herein the construction and content of datasets for the pathology reporting of biopsy and resection specimens of primary bone sarcomas internationally agreed upon by a multidisciplinary group of bone tumour experts working in tertiary referral centres for bone sarcoma are reported. The current evidence was considered and, where lacking, a panel consensus was reached. Data from the relevant medical literature, including the 5^th^ edition of the WHO Classification^5^ as well as other existing published guidelines, were considered.^1^, ^2^, ^3^

The use of standardised reporting templates varies widely. Some pathologists may not engage a standardised template if it is laborious and time‐consuming, and lacks the flexibility desired for providing a more nuanced description of the differential diagnosis.^17^ A survey of pathologists demonstrated that only 44% agreed that standardised reporting facilitates reporting of an accurate diagnosis.^17^ However, it is well established that structured pathology reporting ensures a more complete diagnosis and, as a consequence, improved treatment decisions and patient outcomes.^18^, ^19^, ^20^ Moreover, structured standardised reporting will accommodate cancer registries and facilitate future large‐scale artificial intelligence‐based studies. Standardised reporting is essential for machine actionability, i.e, the capacity of computational systems to find, access, interoperate, and reuse data with minimal human intervention.^21^, ^22^ These so‐called FAIR principles (Findable, Accessible, Interoperable, and Reusable) are especially important for rare cancers such as bone sarcomas, where collaboration in research is often required to achieve significant numbers of patients for meaningful statistical analysis. Worldwide standardised reporting is the first step towards FAIR data registration and stewardship and may enable future distributed machine‐learning approaches for rare bone sarcomas,^23^ where local databases are connected across institutions and countries without the necessity for patient data ever to leave the institute of healthcare provision.^23^ This will unlock research opportunities that are currently prohibited by differences in registration, noncompatibility of information systems, and privacy and regulatory concerns.^23^ The support for standardised reporting can be improved when it is supported by all multidisciplinary team members, when compatibility with other information systems is assured, and when incorporated in speech recognition systems.^17^

In conclusion, we propose here two international datasets for standardised reporting in bone sarcoma care to improve the diagnosis, treatment, and outcome for these patients and to facilitate future machine‐based learning approaches for these rare sarcomas.

## Funding

This research did not receive any specific grant from funding agencies in the public, commercial, or not‐for‐profit sectors.

## Conflict of interest

The authors report no relevant conflicts of interest.

## Author contributions

JVMGB wrote the initial draft with final review and revision by FW, FA, DB, JAB, JLB, JC, EdA, APDT, KJ, AM, GN, AR, AW, AY, and CF.

## Data Availability

Data sharing is not applicable to this article as no new data were created or analyzed in this study.

## References

[1] Royal College of Pathologists . Dataset for histopathological reporting of primary bone tumours. (2021) Available at: https://www.rcpath.org/uploads/assets/83872df2‐d4aa‐4594‐b360b963afb2da51/G096‐Dataset‐for‐histopathology‐reports‐on‐primary‐bone‐tumours.pdf (Accessed 22nd April 2022).

[2] College of American Pathologists . Protocol for the examination of resection specimens from patients with primary tumors of bone. (2021) Available at: https://documents.cap.org/protocols/Bone_4.1.1.0.REL_CAPCP.pdf (Accessed 22nd April 2022).

[3] College of American Pathologists . Protocol for the examination of biopsy specimens from patients with primary tumors of bone. (2021) Available at: https://documents.cap.org/protocols/Bone.Bx_4.1.0.1.REL_CAPCP.pdf (Accessed 22nd April 2022).

[4] Merlin T , Weston A , Tooher R . Extending an evidence hierarchy to include topics other than treatment: revising the Australian 'levels of evidence'. BMC Med. Res. Methodol. 2009; 9; 34. 19519887 10.1186/1471-2288-9-34 PMC2700132

[5] WHO Classification of Tumours Editorial Board . Soft tissue and bone tumours. WHO classification of tumours. Vol. 3. 5th ed. Lyon: IARC Publications, 2020.

[6] Brierley JD , Gospodarowicz MK , Wittekind C . Union for international cancer control. TNM classification of malignant tumours. 8th ed. USA: Wiley, 2016.

[7] Amin MB , Edge S , Greene FL *et al*. AJCC cancer staging manual. 8th ed. New York: Springer, 2017.

[8] Evans HL , Ayala AG , Romsdahl MM . Prognostic factors in chondrosarcoma of bone: a clinicopathologic analysis with emphasis on histologic grading. Cancer 1977; 40; 818–831. 890662 10.1002/1097-0142(197708)40:2<818::aid-cncr2820400234>3.0.co;2-b

[9] Cates JMM . Modeling continuous prognostic factors in survival analysis: implications for tumor staging and assessing chemotherapy effect in osteosarcoma. Am. J. Surg. Pathol. 2018; 42; 485–491. 29200101 10.1097/PAS.0000000000000995

[10] Albergo JI , Gaston CL , Laitinen M *et al*. Ewing's sarcoma: only patients with 100% of necrosis after chemotherapy should be classified as having a good response. Bone Joint J. 2016; 98‐B; 1138–1144. 10.1302/0301-620X.98B8.37346 27482030

[11] Picci P , Rougraff BT , Bacci G *et al*. Prognostic significance of histopathologic response to chemotherapy in nonmetastatic Ewing's sarcoma of the extremities. J. Clin. Oncol. 1993; 11; 1763–1769. 8355043 10.1200/JCO.1993.11.9.1763

[12] van der Woude HJ , Bloem JL , Taminiau AH , Nooy MA , Hogendoorn PC . Classification of histopathologic changes following chemotherapy in Ewing's sarcoma of bone. Skeletal Radiol. 1994; 23; 501–507. 7824975 10.1007/BF00223077

[13] Oberlin O , Deley MC , Bui BN *et al*. Prognostic factors in localized Ewing's tumours and peripheral neuroectodermal tumours: the third study of the French Society of Paediatric Oncology (EW88 study). Br. J. Cancer 2001; 85; 1646–1654. 11742482 10.1054/bjoc.2001.2150 PMC2363978

[14] Milano GM , Cozza R , Ilari I *et al*. High histologic and overall response to dose intensification of ifosfamide, carboplatin, and etoposide with cyclophosphamide, doxorubicin, and vincristine in patients with high‐risk Ewing sarcoma family tumors: the bambino Gesù Children's hospital experience. Cancer 2006; 106; 1838–1845. 16532434 10.1002/cncr.21780

[15] Pan HY , Morani A , Wang WL *et al*. Prognostic factors and patterns of relapse in Ewing sarcoma patients treated with chemotherapy and r0 resection. Int. J. Radiat. Oncol. Biol. Phys. 2015; 92; 349–357. 25772182 10.1016/j.ijrobp.2015.01.022 PMC4431926

[16] Wagner MJ , Gopalakrishnan V , Ravi V *et al*. Vincristine, ifosfamide, and doxorubicin for initial treatment of Ewing sarcoma in adults. Oncologist 2017; 22; 1271–1277. 28710342 10.1634/theoncologist.2016-0464 PMC5634776

[17] Swillens JEM , Sluijter CE , Overbeek LIH , Nagtegaal ID , Hermens R . Identification of barriers and facilitators in nationwide implementation of standardized structured reporting in pathology: a mixed method study. Virchows Arch. 2019; 475; 551–561. 31270615 10.1007/s00428-019-02609-6 PMC6861434

[18] Baranov NS , Nagtegaal ID , van Grieken NCT *et al*. Synoptic reporting increases quality of upper gastrointestinal cancer pathology reports. Virchows Arch. 2019; 475; 255–259. 31144018 10.1007/s00428-019-02586-w PMC6647878

[19] Sluijter CE , van Workum F , Wiggers T *et al*. Improvement of care in patients with colorectal cancer: influence of the introduction of standardized structured reporting for pathology. JCO Clin. Cancer Inform. 2019; 3; 1–12. 10.1200/CCI.18.00104 31070983

[20] Gill AJ , Johns AL , Eckstein R *et al*. Synoptic reporting improves histopathological assessment of pancreatic resection specimens. Pathology 2009; 41; 161–167. 19320058 10.1080/00313020802337329

[21] Wilkinson MD , Dumontier M , Aalbersberg IJ *et al*. The FAIR guiding principles for scientific data management and stewardship. Sci. Data 2016; 3; 160018. 26978244 10.1038/sdata.2016.18 PMC4792175

[22] Jacobsen A , de Azevedo RM , Juty N *et al*. FAIR principles: interpretations and implementation considerations. Data Intell. 2020; 2; 10–29.

[23] Deist TM , Dankers F , Ojha P *et al*. Distributed learning on 20 000+ lung cancer patients ‐ the personal health train. Radiother. Oncol. 2020; 144; 189–200. 31911366 10.1016/j.radonc.2019.11.019

